# Basement membrane ultrastructure and component localization data from uterine tissues during early mouse pregnancy

**DOI:** 10.1016/j.dib.2016.10.033

**Published:** 2016-11-05

**Authors:** Celestial R. Jones-Paris, Sayan Paria, Taloa Berg, Juan Saus, Gautam Bhave, Bibhash C. Paria, Billy G. Hudson

**Affiliations:** a Department of Pathology, Microbiology, and Immunology, Vanderbilt University Medical Center, Nashville, Tennessee, United States; b Aspirnaut, Vanderbilt University Medical Center, Nashville, Tennessee, United States; c Valencia University Medical School, Valencia, Spain; d FibroStatin, SL, Valencia, Spain; e Division of Nephrology and Hypertension, Department of Medicine, Vanderbilt University Medical Center, Nashville, Tennessee, United States; f Center for Matrix Biology, Vanderbilt University Medical Center, Nashville, Tennessee, United States; g Division of Neonatology, Department of Pediatrics, Vanderbilt University Medical Center, Nashville, Tennessee, United States; h Department of Biochemistry, Vanderbilt University, Nashville, Tennessee, United States; i Department of Cell and Developmental Biology, Vanderbilt University Medical Center, Nashville, Tennessee, United States; j Vanderbilt Ingram Cancer Center, Nashville, Tennessee, United States; k Vanderbilt Institute of Chemical Biology, Nashville, Tennessee, United States

**Keywords:** Basement membrane

## Abstract

Basement membranes (BMs) are specialized extracellular scaffolds that provide architecture and modulate cell behaviors in tissues, such as fat, muscle, endothelium, endometrium, and decidua. Properties of BMs are maintained in homeostasis for most adult tissues. However, BM ultrastructure, composition, and localization are rapidly altered in select uterine tissues that are reprogrammed during pregnancy to enable early maternal-embryo interactions. Here, our data exhibit both static and dynamic BMs that were tracked in mouse uterine tissues during pre-, peri-, and postimplantation periods of pregnancy. The data exhibit spatial-temporal patterns of BM property regulation that coincide with the progression of adapted physiology. Further interpretation and discussion of these data in this article are described in the associated research article titled, “Embryo implantation triggers dynamic spatiotemporal expression of the basement membrane toolkit during uterine reprogramming” (C.R. Jones-Paris, S. Paria, T. Berg, J. Saus, G. Bhave, B.C. Paria, B.G. Hudson, 2016) [1].

**Specifications Table**

**Table:** 

Subject area	Biology
More specific subject area	Matrix Biology during Uterine Physiology for Pregnancy
Type of data	Image (microscopy)
How data was acquired	Brightfield microscope (Olympus Bx51 with DP72 camera), Transmission electron microscope (Philips/FEI T-12); High-resolution immunofluorescence automated microscope (Leica Ariol SL-50 or Apiro Versa 200)
Data format	Raw, processed
Experimental factors	Physiological responses to pregnancy; pseudopregnancy reaction; Collected uterine tissues that were processed for microscopy-based analysis
Experimental features	Histological staining; electron micrographs; immunofluorescence
Data source location	Nashville, Tennessee, United States
Data accessibility	Data is with this article.

**Value of the data**

• These data of H&E stainings and schematics are useful for researchers interested in tissue and cellular-level histological changes that occur in mouse uterine tissues during early pregnancy.
• The presented electron micrographs provide a context of cells and basement membrane for interpretation of cell–matrix and cell–cell orientation and interactions.
• The immunofluorescence data include entire cross-sections through uterine tissues that allow comparative analysis of dynamic and homeostatic localization of basement membrane components during pregnancy.
• The positive and negative control data provide evidence of immunofluorescence detection specificity and are informative to researchers that apply the same antibodies in future experiments.

## 1 Data

**Fig. 1:**
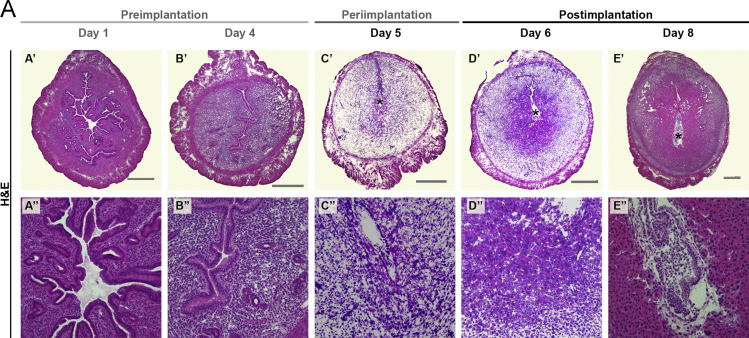
Histological examples of uterine tissue organization during early pregnancy. Brightfield micrographs of H&E-stained mouse uterus during early pregnancy. Scale bar=500 μm. Blue inset squares in A’–E’ are indicative of the field of view in A”–E”. Asterisk (^⁎^) indicates the location of the embryo.

**Fig. 2:**
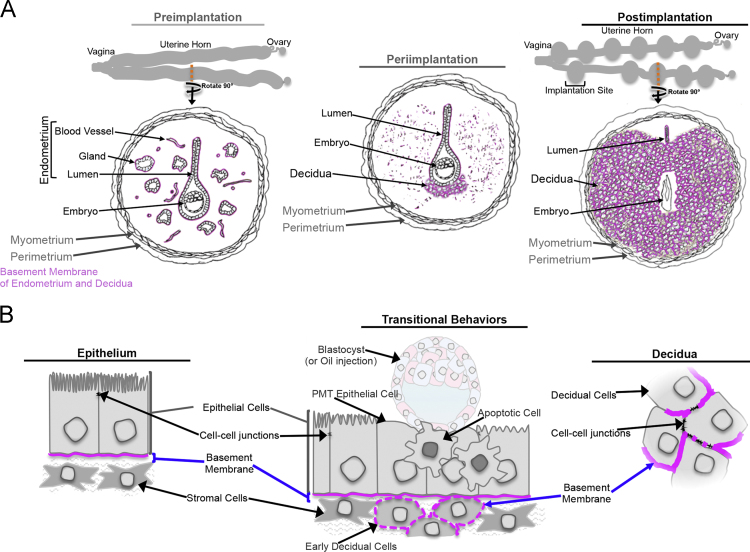
Diagrams of uterine tissue anatomy and basement membrane arrangement during early pregnancy. **A)** Schematic of BM localization during tissue-level adapted physiology of uterus during early mouse pregnancy. **(B)** Schematic of BM localization during cell-level adapted physiology of uterus during early mouse pregnancy. PMT, plasma membrane transformation. Pre-, peri-, and postimplantation stage changes exemplified and basement membranes are represented by pink patterns in **(A)** and **(B)**.

**Fig. 3:**
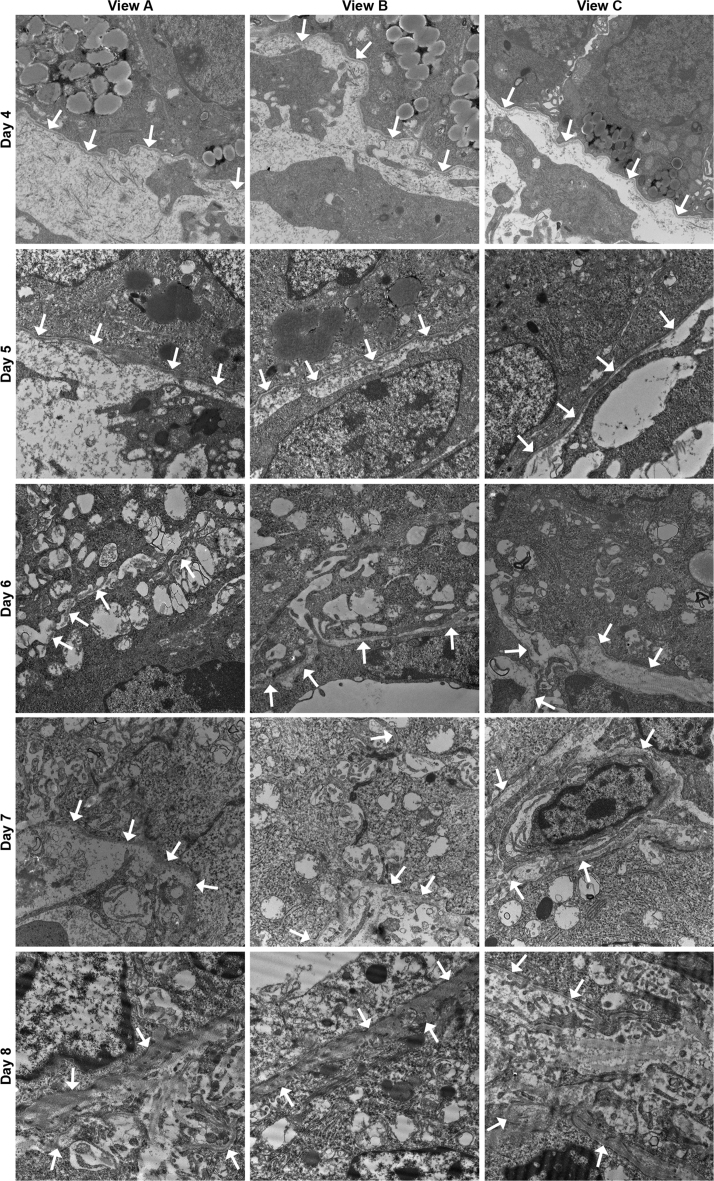
Early pregnancy basement membrane ultrastructure in endometrium and decidua. Transmission electron microscopy 15000x magnification micrographs of mouse uterus day 4–8 of pregnancy with pericellular lamina densa (basement membrane) represented in Fig. 1, uncropped. Arrows point toward regions of lamina densa.

**Fig. 4:**
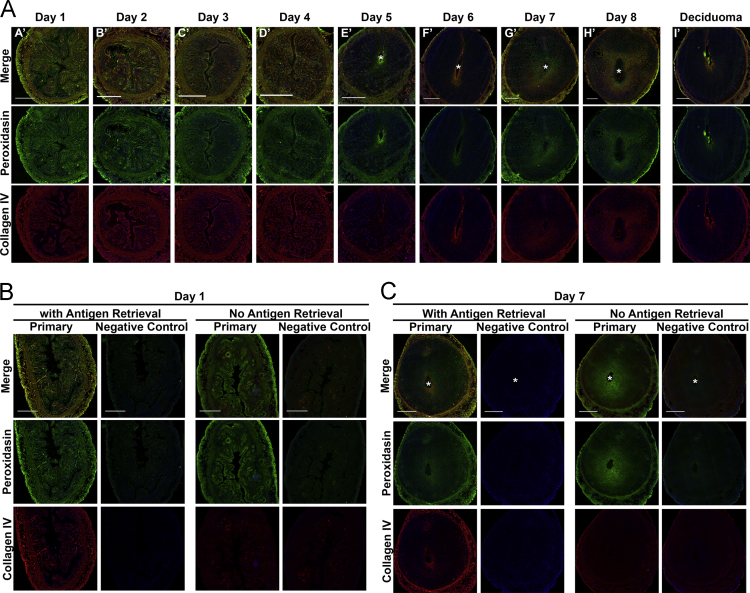
Collagen IV and peroxidasin localization during early pregnancy. **A)** Full cross-section of mouse uterus double immunofluorescence of collagen IV and peroxidasin during early pregnancy with collagen IV in red, peroxidasin in green, and nuclei in blue. Co-localization of collagen IV and peroxidasin is indicated by orange/yellow color. Asterisk (^⁎^) indicates the location of the embryo. Scale bar=500 μm. Representative immunofluorescence controls preimplantation **(B)** and postimplantation **(C)**.

**Fig. 5:**
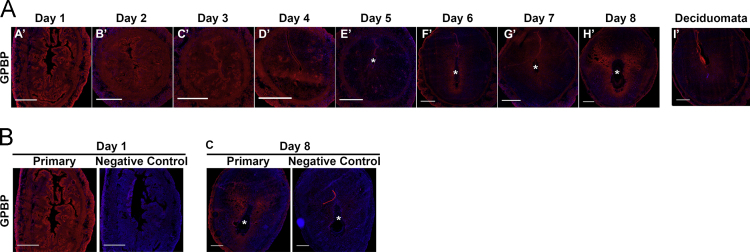
Localization of GPBP during early pregnancy. **A)** Full cross-section of mouse uterus immunofluorescence of GPBP during early pregnancy with GPBP in red and nuclei in blue. Asterisk (^⁎^) indicates the location of the embryo. Scale bar=500 μm. Representative immunofluorescence controls preimplantation **(B)** and postimplantation **(C)**.

**Fig. 6:**
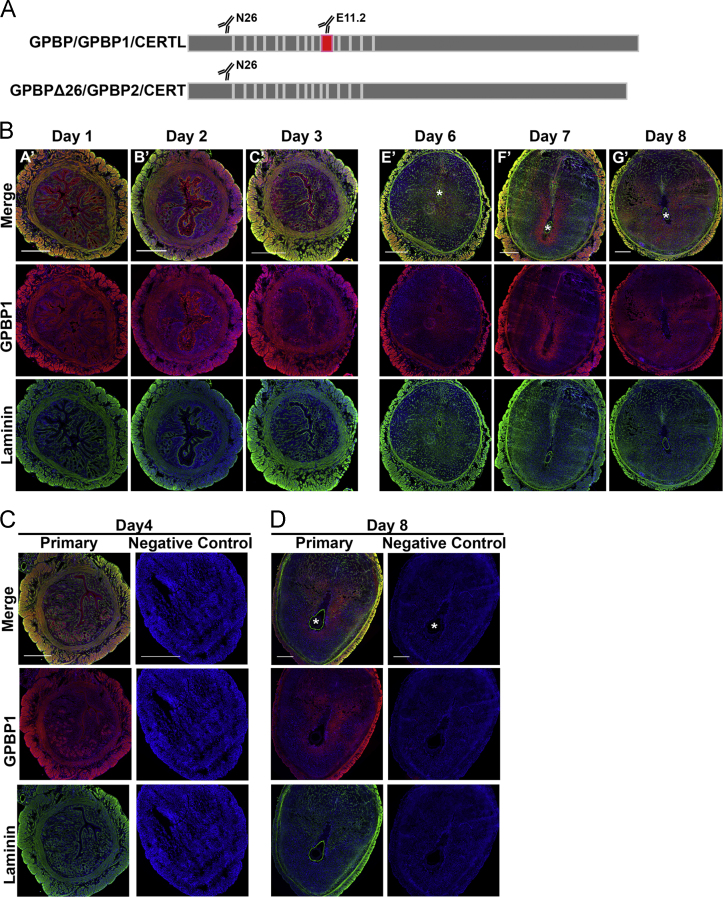
Isoform GPBP-1 and laminin localization during early pregnancy. **A**) Diagram depicting areas of GPBP isoforms that mAb N26 and e11-2 specifically bind. The N26 antibody recognizes all isoforms of GPBP while the e11-2 antibody detects the 26-amino acid region that is encoded by exon 11 (shown as the red block), which is not present in GPBP-2 also called GPBPΔ26 or CERT. **B**) Full cross-section of mouse uterus double immunofluorescence of GPBP-1 and laminin during early pregnancy with GPBP-1 in red, laminin in green, and nuclei in blue. Colocalization of GPBP-1 and laminin is indicated by orange/yellow color. Asterisk (^⁎^) indicates the location of the embryo. Scale bar=500 μm. Representative immunofluorescence controls preimplantation **(C)** and postimplantation **(D)**.

**Fig. 7:**
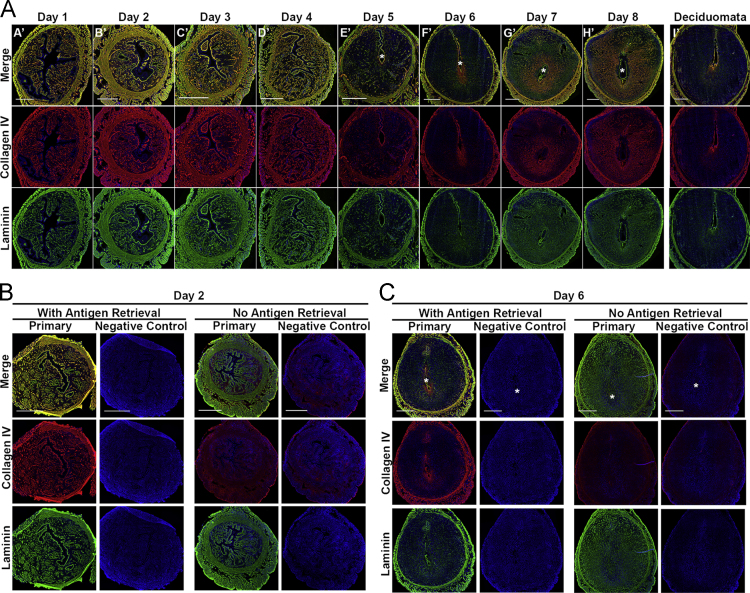
Collagen IV and laminin localization during early pregnancy. **A)** Full cross-section of mouse uterus double immunofluorescence of collagen IV and laminin during early pregnancy with collagen IV in red, laminin in green, and nuclei in blue. Colocalization of collagen IV and laminin is indicated by orange/yellow color. Asterisk (^⁎^) indicates the location of the embryo. Scale bar=500 μm. Representative immunofluorescence controls preimplantation **(B)** and postimplantation **(C)**.

The data in this article include hematoxylin and eosin (H&E) histological visualization of uterine tissue changes that naturally occur during early mouse pregnancy (Fig. 1). A schematic (Fig. 2) (included in part in [1]) is presented for anatomical spatial orientation of dynamic basement membranes (BMs) that is beneficial in cross-examination of BM ultrastructure (Fig. 3) and localization of individual components: peroxidasin (Fig. 4); GPBP (Fig. 5); GPBP-1, a variant of GPBP that can be trafficked to extracellular spaces (Fig. 6); and collagen IV and laminin (Fig. 7).

## 2 Materials and methods

### 2.1 Animals

Healthy adult CD1 mice (Charles River Laboratories, Crl:CD1(ICR), Wilmington, MA, USA) were bred and the presence of a vaginal plug the following morning was established as day 1 of pregnancy [2]. Uterine horns were harvested on days 1 through 4 of pregnancy. Chicago Blue 6B dye (Sigma, St. Louis, MO) was used to distinguish implantation sites on day 5 of pregnancy with methods previously described [3]. Implantation sites from days 6 through 8 of pregnancy were collected by anatomical identification. The deciduomata, a decidual reaction triggered by non-embryonic experimental stimulus, was induced by intraluminal injection sesame oil into the uterine horn on day 4 of pregnancy then tissues were collected 48 h post injection as described [4]. Uterine samples were collected in the mornings on designated days of pregnancy, snap frozen, and stored at −80 °C. All animal procedures were conducted in accordance with guidelines and standards of the Society for the Study of Reproduction (SSR) and approved by Vanderbilt University׳s Institutional Animal Care and Use Committee (IACUC).

### 2.2 Histochemical staining and Immunofluorescence

Uterine samples were embedded in Optimal Cutting Temperature (OCT) compound (Sakura Tissue-Tek, Torrance, CA), cryosectioned (Leica Biosystems, Buffalo Grove, IL), and collected onto slides (Fisher Scientific, Waltham, MA). Samples were processed and stained with hematoxylin and eosin (H&E) using routine methods at the Vanderbilt University Medical Center Translational Pathology Shared Resource (TPSR, Nashville, TN). Briefly, samples were carefully acclimated to room temperature, washed with phosphate-buffered saline (PBS) for 3 min to remove OCT, fixed with 10% neutral buffered formalin for 3 min, and rinsed with running water. Staining was carried out by incubating in hematoxylin and then eosin by dipping 15 times in each dye, rinsing with running water between dyes. Sections were then dehydrated in 3 exchanges of ethanol and 2 exchanges of xylene and mounted in non-aqueous media with a coverslip.

Cryosections processed for immunofluorescence were air-dried and fixed in −20 °C acetone (Sigma, St. Louis, MO) then were washed several times with phosphate-buffered saline (PBS) (Corning, Corning, NY) and PBS with 0.2% Tween (Sigma, St. Louis, MO). Nonspecific binding was blocked by incubation of samples with 10% normal goat or horse serum (Invitrogen, Grand Island, NY). All antibodies were diluted in PBS/0.1% Tween with 5% goat serum. The following primary antibodies were applied to the sections: rat anti-collagen IV NC1 (1:500 dilution, JK2, were from Y. Sado, Shigei Medical Research Institute, Okayama, Japan [5], [6]), rabbit anti-laminin (1:50 dilution for tissues not treated with dissociation buffer and 1:500 dilution for those that were pre-treated with dissociation buffer, ab11575, Abcam, Cambridge, MA), rabbit anti-peroxidasin (1:250 dilution, were from G. Bhave, Vanderbilt University, Nashville, TN [7]), Alexa546 conjugated mouse anti-GPBP (1:50 dilution, mAb N26 to the N-terminal serine-rich domain conserved across all GPBP isoforms in several species [8], from Fibrostatin, SL, Valencia, Spain), and Alexa546 conjugated mouse anti-GPBP-1 (1:50 dilution, mAb e11-2 to the 26-amino acid residue of exon 11 not present in GPBP-2, also called CERT [9], from Fibrostatin, SL, Valencia, Spain). A previously described dissociation buffer was used on sections co-stained with collagen IV antibody (JK2) [1], [5] followed by several washes with PBS and PBS/0.2% Tween. Detection and co-detection with GPBP antibodies (mAbs N26 and e11-2) were performed without dissociation treatment. Sections were washed with PBS/0.2% Tween before applying the following secondary antibodies: Alexa555 goat anti-rat (1:200 dilution, ab150166, Abcam) and Alexa488 goat anti-rabbit (1:200 dilution, ab150081, Abcam). Nuclei were stained with Hoechst dye. Following several washes, sections were mounted in Prolong Gold (Life Technologies, Grand Island, NY). After mounting solution cured, sections were imaged with 20x objective on a automated microscope (Ariol SL-50 or Apiro Versa 200 Leica Biosystems, Buffalo Grove, IL) at the Vanderbilt Univeristy Medical Center Digital Histology Shared Resource (Nashville, TN). Consistent Linear adjustments were applied to brightness and contrast of images with settings established relative to representative controls.

### 2.3 Transmission Electron Microscopy (TEM)

Fresh uterine tissues were fixed in glutaraldehyde with sodium cacodylate. Then, samples were further processed in the Vanderbilt University Medical Center Cell Imaging Shared Resource. Briefly, tissues underwent several exchanges of fixative and dehydrating solvents. Samples were then infiltrated gradually with epoxy resin (ER). Then samples were cured [10]. Thin sections were cut using an ultramicrotome (Leica ultracut, Buffalo Grove, IL) and then mounted onto copper grids. The sections were stained with uranyl acetate and Reynold lead citrate before they were imaged at 30,000x on an electron microscope (Philips T-12 equipped with an AMT CCD camera system, FEI, Hillsboro, OR). Further details of immunofluorescence and electron microscopy methods are available in the accompanying research article [1].

## Conflict of interest

The authors declare no conflicts of interest.
